# Clinical and Imaging Predictors of Surgical Outcome in Multilevel Cervical Ossification of Posterior Longitudinal Ligament: An Analysis of 184 Patients

**DOI:** 10.1371/journal.pone.0136042

**Published:** 2015-09-01

**Authors:** Yifei Gu, Jueqian Shi, Peng Cao, Wen Yuan, Huiqiao Wu, Lili Yang, Ye Tian, Lei Liang

**Affiliations:** 1 Department of Spine Surgery, Changzheng Hospital, Second Military Medical University, Shanghai, China; 2 Department of Radiology, Shanghai Chest Hospital, Shanghai, China; University of Toronto, CANADA

## Abstract

**Objective:**

To investigate the clinical and imaging predictors of surgical outcomes in patients with ossification of the posterior longitudinal ligament (OPLL).

**Materials and Methods:**

From May 2010 to April 2012, a total of 200 consecutive patients with cervical OPLL were recruited for this study. Of them, 184 patients (130 men and 54 women) who could be tracked for more than 24 months after surgery were finally included for analysis. Their demographic, clinical and radiological data were collected preoperatively. The recovery ratio in terms of JOA score was used to assess the outcome of the patients preoperatively and at 2 years postoperatively. A JOA recovery rate less than 50% was considered a poor outcome.

**Results:**

Compared with good outcome group, an older mean age at operation, a longer mean duration of symptoms, a lower mean pre-operativer JOA score, and a higher proportion of diabetics were observed in poor outcome group. Patients in poor outcome group were more likely to present kyphotic cervical alignment, smaller mean transverse area of the spinal cord, and intramedullary signal abnormalities. The result of multivariate stepwise logistic regression showed that a longer duration of symptoms and the presence of T1 hypo-intensity intramedullary changes on MRI were significant risk factors of lower JOA recovery ratios.

**Conclusion:**

A longer duration of symptom, T1 hypointensity on MRI and a history of minor trauma were highly predictive of a poor outcome for patients undergoing surgical treatment of OPLL. Age at operation, the history of diabetes, the preoperative JOA score, the transverse area of the spinal cord and T2 hyper-intensity on MRI were also associated with the prognosis of OPLL.

## Introduction

Cervical myelopathy due to ossification of the posterior longitudinal ligament (OPLL) is a common cause of spinal cord dysfunction[1]. As persistent compression of the spinal cord by OPLL may lead to severe neurological deterioration for which conservative therapy has proved to be ineffective, surgical treatment is often necessary in most cases. Although various surgical strategies including anterior decompression and posterior decompression have proved to be mature techniques, unsatisfied outcomes and associated complications are not uncommon[2–8].

Knowing that prediction is valuable in helping determine what is the optimal time of surgical intervention in the course of disease progression and what patients are most likely to have positive response to surgery, it is important to know which prognostic factors are most predictive of satisfactory surgical outcomes. There have been several studies concerning the correlation between different characteristics of patients and the outcome of surgical treatment of OPLL[9–11]. However, most of them only used univariate analysis to estimate the prognosis. Given great differences in the epidemiology and anatomy between individual patients, a prospective multivariate analysis is needed to exclude confounding factors. The aim of this multivariate analysis is to identify the key clinical and imaging characteristics that can help predict the outcome of patients undergoing surgical treatment for OPLL.

## Methods

### Ethics statement

This study was approved by the Ethics Committee of Changzheng Hospital (Shanghai, China). All subjects provided free written informed consent. Research was conducted in accordance with the research principles in the Declaration of Helsinki.

### Patients population

A total of 200 consecutive patients with cervical OPLL who were referred for surgical treatment in our department between May 2010 and April 2012 were recruited for this study. The clinical diagnosis of cervical OPLL was confirmed by CT and MRI examinations in all patients who failed to respond to nonsurgical treatment. Exclusion criteria were patients with malignancies, histories of cervical spine surgery, and major traumatic cord injuries with cervical laminar fractures, bony fractures, or dislocations caused by high-energy trauma. Patients with confirmed myeloradiculopathy due to lumbar or thoracic compression or other diseases that may cause sensory and/or motor disturbances such as cerebral infarction, arteritis and joint osteoarthritis were also excluded.

Anterior cervical corpectomy and fusion (ACCF) was used for cases with the segmental or circumscribed type that did not exceed four intervertebral levels (maximum 3-level corpectomies), or posterior laminoplasty would be used in patients without intervertebral instability, and laminectomy with fixation in patients with intervertebral instability.

Sixteen patients were lost to follow-up, of whom one patient died of an unrelated disease. The remaining 184 patients who could be tracked for more than 24 months after surgery were finally included for analysis.

### Clinical Data Collection

Demographic and clinical data were collected in all patients preoperatively, including age, gender, body mass index (BMI), the history of minor cervical trauma, alcohol and tobacco use, the history of diabetes, and the pre-operative Japanese Orthopedic Association (JOA) score as the clinical predictive factors.

### Imaging assessment

All the enrolled patients underwent X-ray radiography, CT and MRI scans preoperatively. A radiologist who was blinded to the clinical and neurological status of the patients analyzed all radiological parameters as follows.

#### Cervical alignment

**Fig 1:**
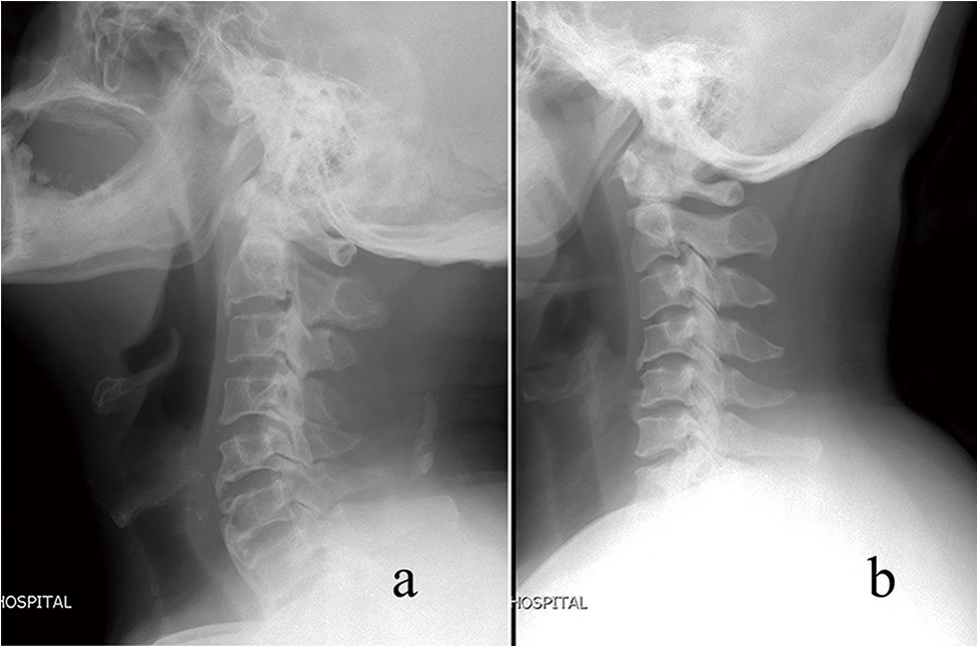
The cervical aliment was classified as lordotic (a) and kyphotic (b).

The C2-7 Cobb angle (α) was measured on the lateral radiograph. The cervical aliment was classified as lordotic (α > 0°) and kyphotic (α < 0°) and sigmoid (Fig 1).

#### Morphological features of OPLL

**Fig 2:**
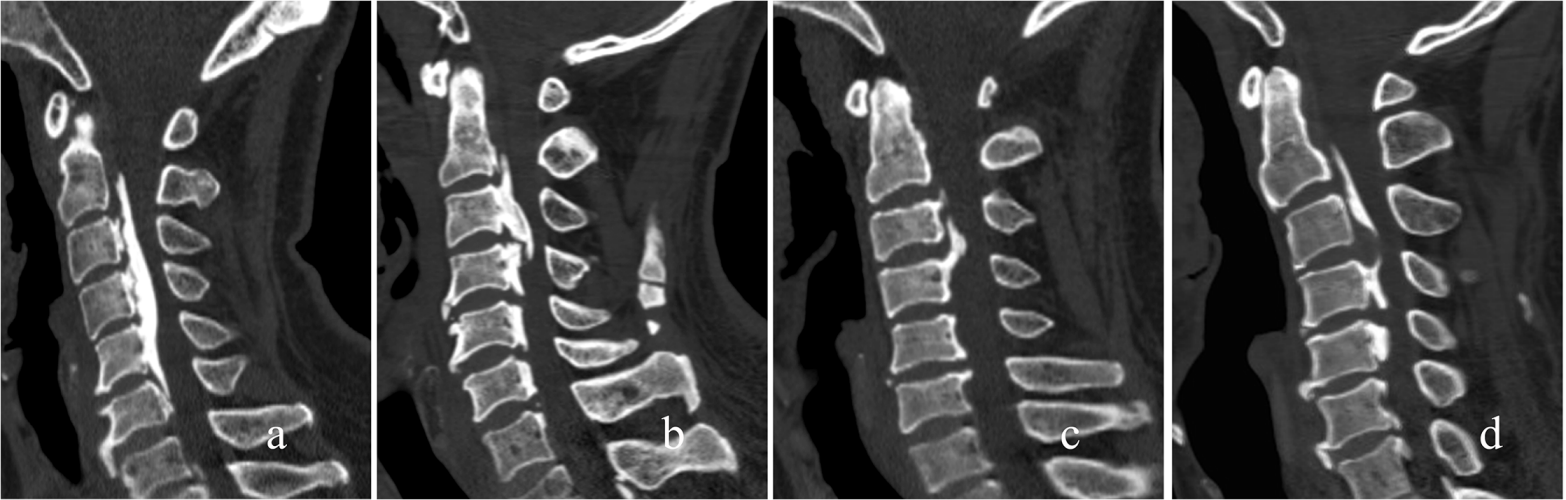
Morphological types of OPLL were classified into the continuous type (a), segmental type (b), circumscribed type (c) and mixed type (d) according to Hirabayashi's classification.

**Fig 3:**
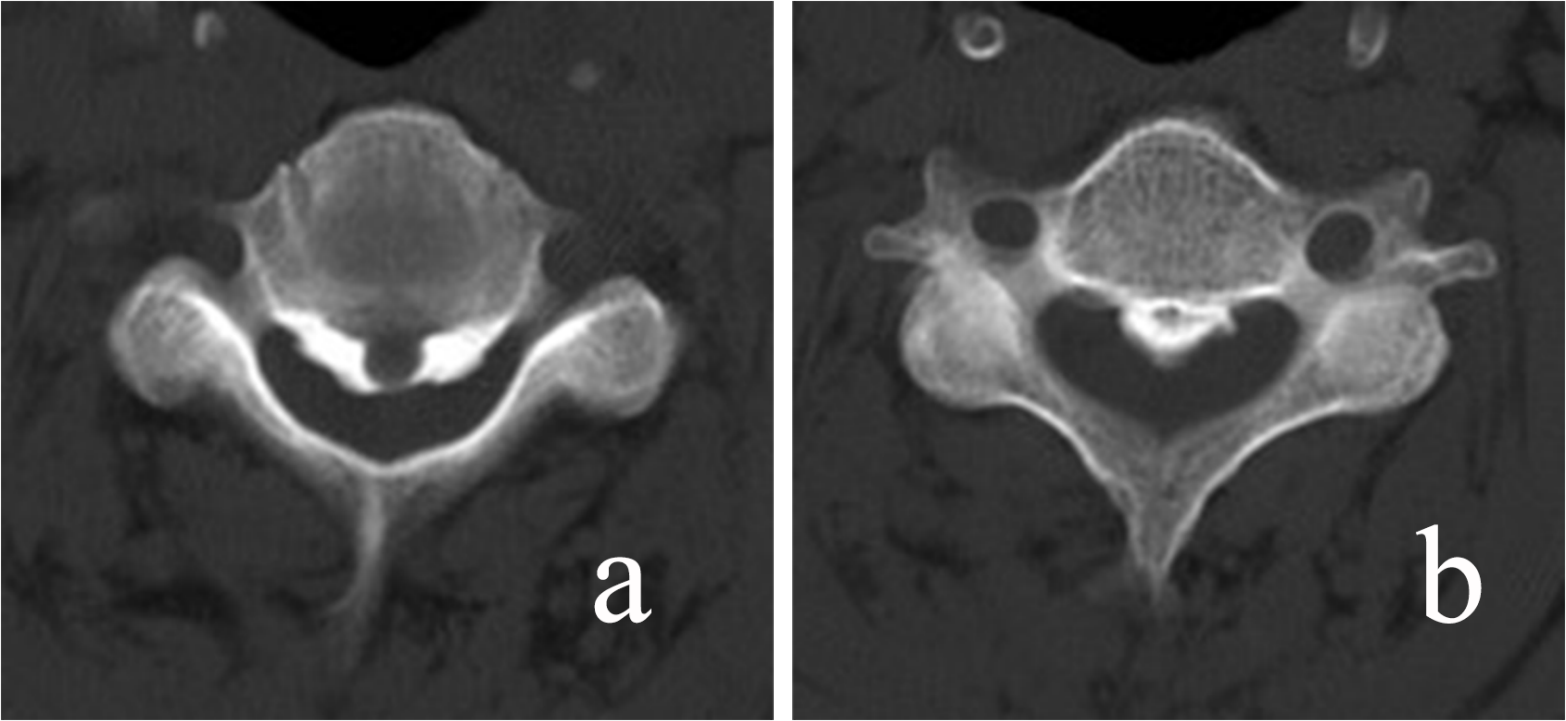
The shape of ossification was defined as the wide-base type and narrow-base type on CT axial imaging.

Morphological types of OPLL were classified as the continuous type, segmental type, circumscribed type and mixed type according to the classification by Hirabayashi et al [12] (Fig 2). The shape of ossification was defined as the wide-base type and narrow-base type on CT axial imaging, and plateau-shaped and hill-shaped on sagittal imaging (Fig 3).

#### Occupying ratio

**Fig 4:**
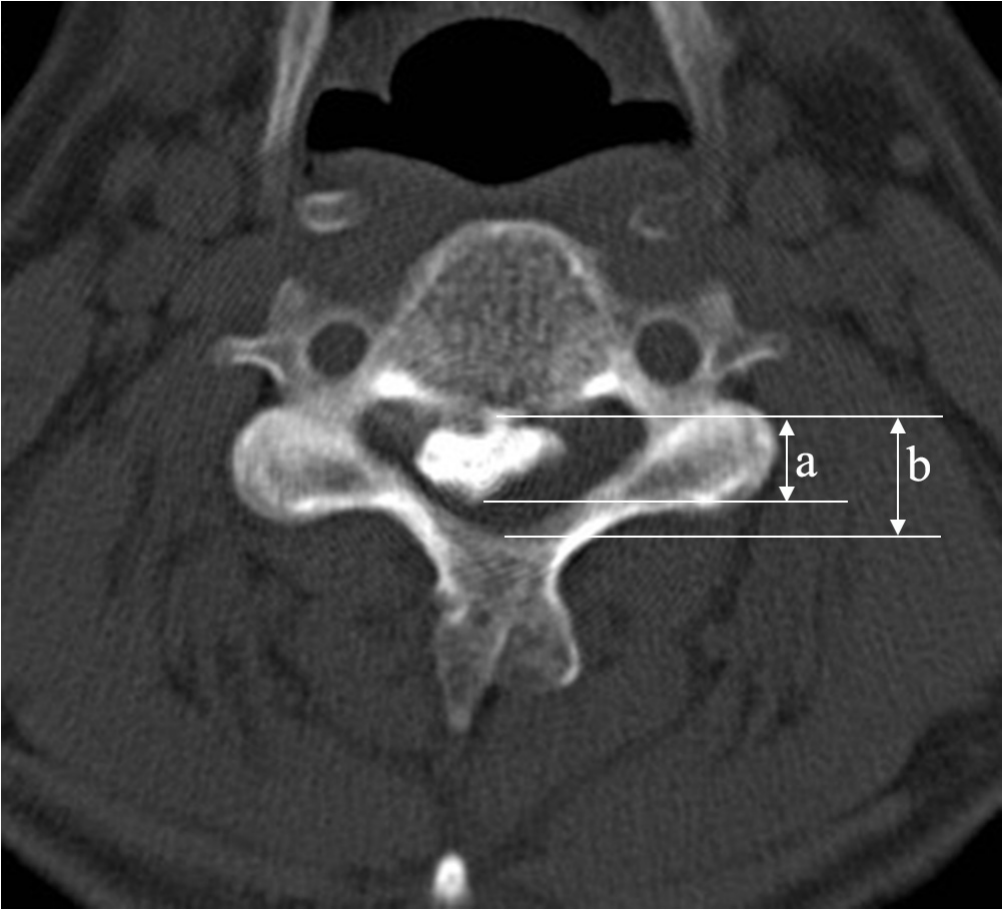
The occupying ratio of the spinal canal was defined as the ratio of the maximal ossification thickness (a) to the anterioposterior spinal canal diameter (b) on CT axial imaging.

The occupying ratio of the spinal canal was defined as the ratio of the maximal ossification thickness to the anterioposterior spinal canal diameter on the CT axial imaging (Fig 4).

#### K-line

**Fig 5:**
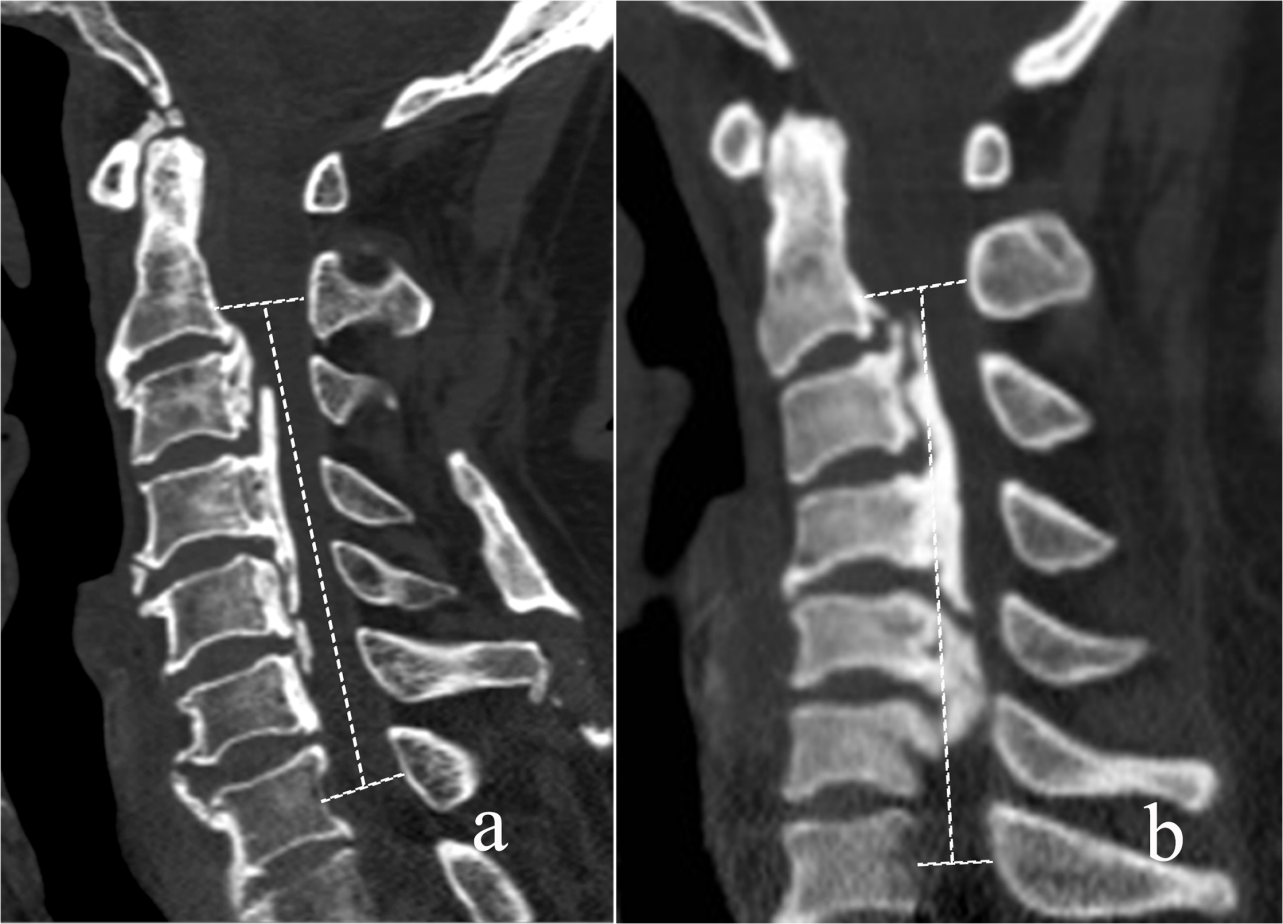
The K-line is a straight line that connects the midpoints of the spinal canal at C2 and C7 on the lateral cervical radiographs. Patients without OPLL exceeding the line were considered as K-line (+) ones (a) and those does exceed it were considered as K-line (-) ones (b).

According to Fujiyoshi et al [13], the K-line is a straight line connecting the midpoints of the spinal canal at C2 and C7 on the lateral cervical radiographs. Patients without OPLL exceeding the K-line were considered as K-line (+) group and those who did not exceed it were considered as K-line (-) group (Fig 5).

#### Spinal cord parameters

**Fig 6:**
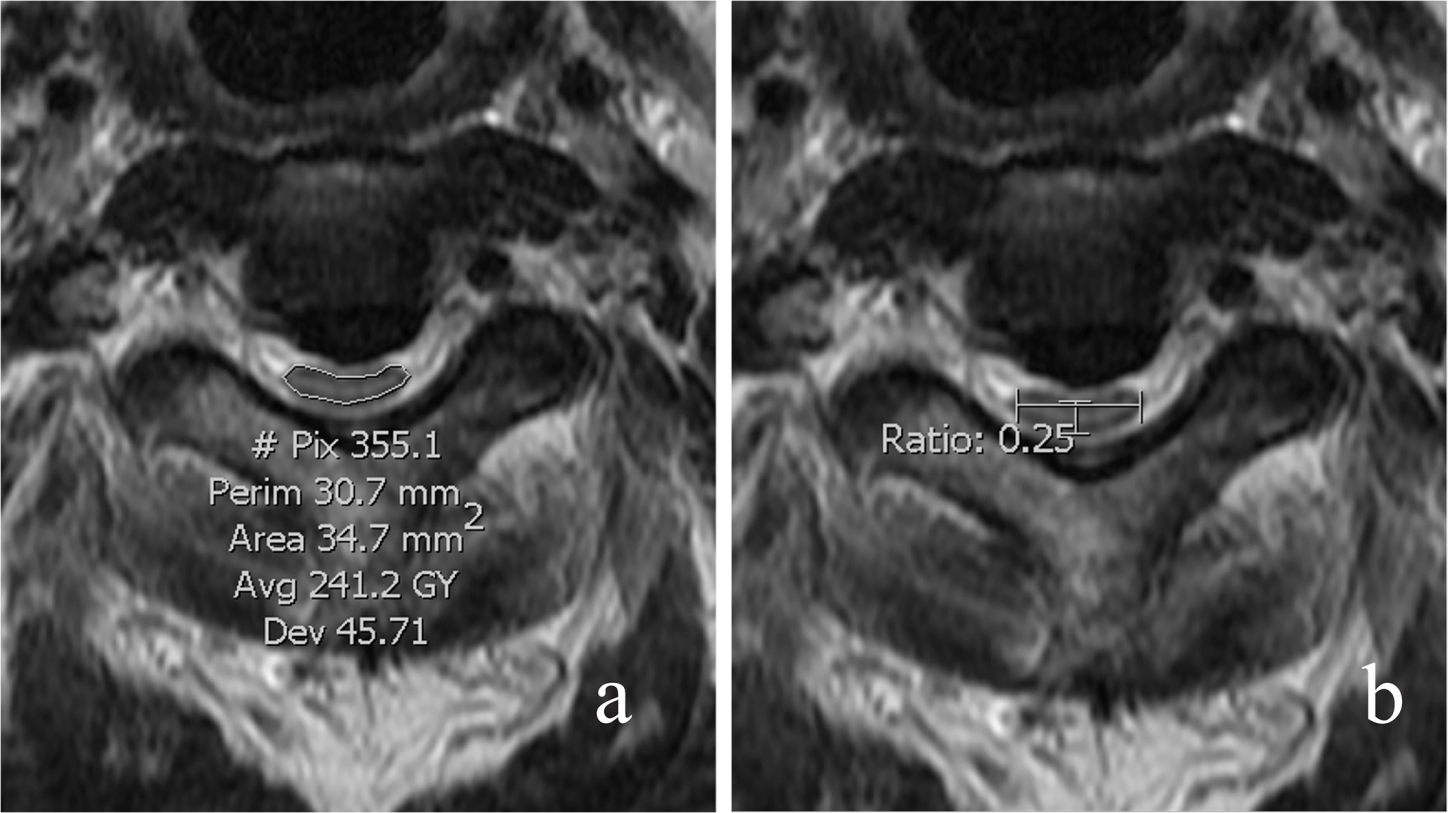
The cross-sectional area of the spinal cord was measured on MR axial imaging at the most compressed segment. The compression ratio of the spinal cord was measured as the ratio of anteroposterior diameter to transverse diameter of the spinal cord on MR axial imaging at the most compressed segment.

**Fig 7:**
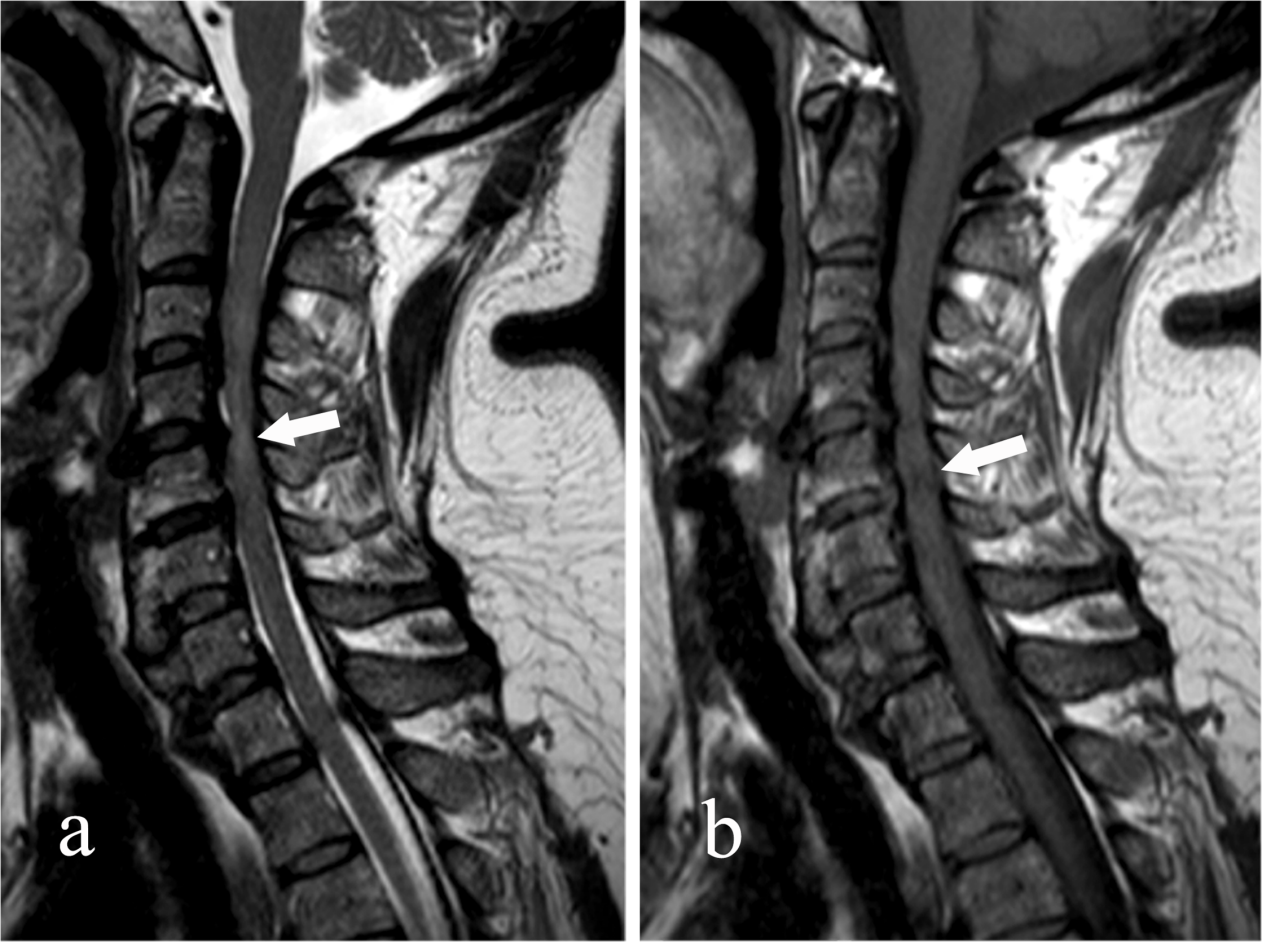
Intramedullary changes in signal intensity on both T1-weighted imaging (T1WI) and T2-weighted imaging (T2WI) of MRI were assessed. Fig 7a shows a T2 hyper-intensity intramedullary change (arrow) and Fig 7b shows a T1 hypo-intensity intramedullary change (arrow) on saggital MRI scan.

The number of levels of compression was assessed on MR saggital imaging. At the most compressed level, the cross-sectional area, the anteroposterior diameters and the transverse diameter of the spinal cord were measured on axial imaging. Compression ratio of the spinal cord = anteroposterior diameter / transverse diameter (Fig 6). Intramedullary change of signal intensity on both T1-weighted imaging (T1WI) and T2-weighted imaging (T2WI) of MRI were assessed (Fig 7).

#### Dural ossification

**Fig 8:**
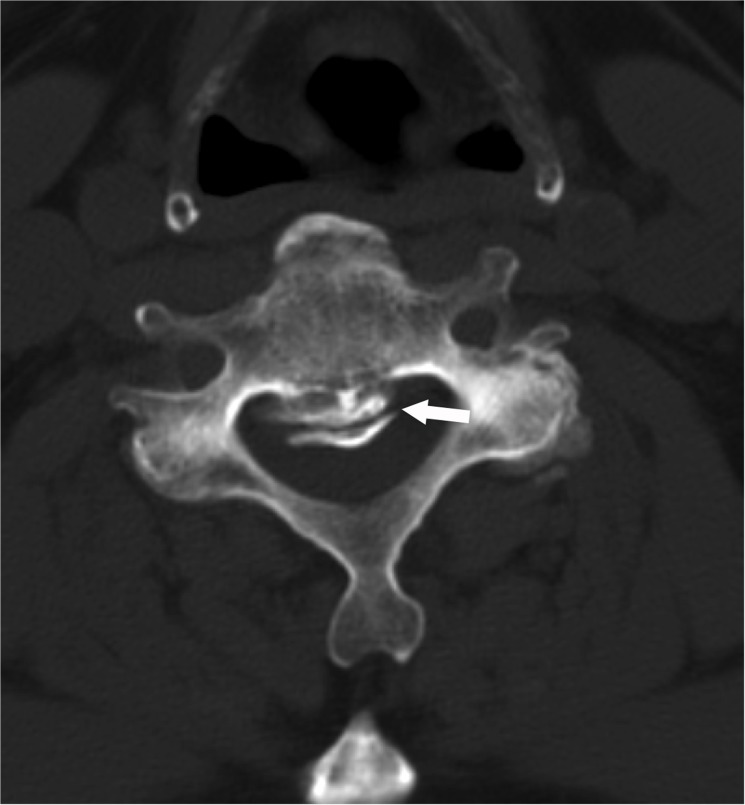
Dural ossification was assessed by the presence of the double-layer sign (arrow).

Dural ossification was assessed by the presence of the double-layer sign[14] (Fig 8).

#### Hypertrophic ligamentum flavum

The presence of simultaneous compression of hypertrophic ligamentum flavum was also assessed on MRI.

### Outcome measures

The recovery ratio in terms of JOA score was used to assess the outcome of patients preoperatively and at 2 years postoperatively. The recovery ratio was evaluated by the Hirabayashi's formula[12]:

(Postoperative JOA score-preoperative JOA score)/(17-preoperative JOA score)×100%

A recovery rate in the JOA less than 50% was considered a poor outcome.

Post-operative complications including hardware failure, cerebrospinal fluid leakage, iatrogenic neurological deterioration and axial pain were recorded as well.

### Statistical analysis

Data were analyzed using the SPSS version 20 software package (IBM SPSS Statistics 20.0, IBM Corporation, Armonk, NY). The mean values are presented as mean ± standard deviation. Intergroup comparisons were made using Wilcoxon rank sum test or Pearson’s χ^2^ test. Risk factors associated with surgical outcomes were identified by the multivariate logistic regression analysis with odds ratios and a 95% confidence interval. A P value < 0.05 was considered statistically significant.

## Results

There were 130 men and 54 women with a mean age of 53.46 (28–81) years. ACCF was performed in 39 patients, laminectomy and fusion in 98 patients, and laminoplasty in 47 patients. The demographic and diagnostic characteristics of the included patients are presented in Table 1.

**Table 1:** Descriptive characteristics of the patient population.

Characteristics	
Patients (n)	184
Age at operation (year)	53.46±9.701
Male/Female	130/54
Duration of symptoms (month)	41.15± 41.290
Follow-up period	27.39±9.463
Surgical approach (n)	
ACCF	39 (21.2%)
Laminoplasty	98 (53.3%)
Laminectomy and fusion	47 (25.5%)
Type of OPLL (n,%)	
Continuous	46 (25%)
Segmental	54 (29.3%)
Mixed	84 (45.7%)
Compression Levels (2/3/4/5/6 levels)	9/33/72/61/9
Pre-OP JOA score	9.59±1.772
Post-OP JOA score	14.64±2.012
Recover Ratio of JOA score (%)	70.52±23.379

The mean JOA score improved from preoperative 9.59±1.772 points to 14.64±2.012 points at 24 months post-operation (P < 0.001). The mean recovery ratio of JOA score was 70.52±23.379%. Adequate cord decompression was achieved in all patients as confirmed by MRI, and none of them required revision surgery.

According to different JOA recovery ratios, patients were classified as good outcome group (n = 135) and poor outcome group (n = 49). Comparison of the patients between the two groups suggested a correlation between certain risk factors and the post-operative outcome. An older mean age at operation, a longer mean duration of symtoms, a lower mean pre-operativer JOA score, and higher proportions of diabetics were observed in poor outcome group. Patients in poor outcome group were more likely to have a kyphotic cervical alignment, smaller mean transverse area of the spinal cord, and intramedullary signal abnormalities (both T1 hypointensity and T2 hyperintensity on MRI) (Table 2).

**Table 2:** Comparison of characteristics of patients with different recovery ratios of JOA score.

Characteristics	Poor Outcome (JOA RR< 50%, n = 49)	Good outcome (JOA RR≥50%, n = 135)	Test value	P value
Sex			χ^2^ = 0.352	0.553
Male	33 (67.3%)	97 (71.9%)		
Female	16 (32.7%)	38 (28.1%)		
Age at operation (years)	57.53±8.775	51.98±9.627	Z = 3.362	**0.001**
Duration of symptoms (months)	68.61±58.244	31.19±27.153	Z = 5.963	**<0.001**
Follow-up period	26.33±5.588	27.78±10.514	Z = 0.836	0.403
History of minor trauma	26 (53.1%)	23 (17.0%)	χ^2^ = 18.112	**<0.001**
History of diabetes (n)	16 (32.7%)	20 (14.8%)	χ^2^ = 7.269	**0.007**
History of smoking (n)	14 (28.6%)	46 (34.1%)	χ^2^ = 0.495	0.482
Pre-JOA score	8.81±1.831	9.81±1.702	Z = -2.945	**0.003**
Post-JOA score	12.20±1.060	15.52±1.475	Z = -9.391	**<0.001**
Type of OPLL			χ^2^ = 0.762	0.683
Continuous	13 (26.5%)	33 (24.4%)		
Segmental	12 (24.5%)	42 (31.1%)		
Mixed	24 (49.0%)	60 (44.4%)		
Shape of OPLL (Sagittal view)				0.095
Hill-shaped	23 (46.9%)	82 (60.7%)		
Plateau-shaped	26 (53.1%)	53 (39.3%)		
Shape of OPLL (Transverse view)			χ^2^ = 0.017	0.898
Symmetrical	34 (69.4%)	95 (70.4%)		
Asymmetrical	15 (32.7%)	40 (29.6%)		
Cervical alignment				
Lordosis	34 (69.4%)	112 (83.0%)	χ^2^ = 4.043	**0.044**
Kyphosis	15 (30.6%)	23 (17.0%)		
Compression levels				
2 /3 /4 /5 /6 levels	2/9/22/14	7/24/50/47	χ^2^ = 1.176	0.882
K-line (-)	3	5	χ^2^ = 0.506	0.477
Occupying ratio of spinal canal (%)	52.63±14.215	50.64±12.691	Z = 1.123	0.261
Transverse area of spinal cord (mm^2^)	51.98±13.838	58.51±16.786	Z = -2.257	**0.024**
Compression ratio of spinal cord (%)	27.27±9.271	26.24±7.024	Z = 0.572	0.567
T1 hypointensity on MRI	16 (32.7%)	104 (77.0%)	χ^2^ = 31.220	**<0.001**
T2 hyperintentsity on MRI	46 (93.9%)	94 (69.6%)	χ^2^ = 11.618	**0.001**
Instability of cervical spine	7 (14.3%)	9 (6.7%)	χ^2^ = 2.629	0.105
Double-layer sign	11 (22.4%)	24 (17.8%)	χ^2^ = 0.509	0.475
Hypertrophy of ligamentum flavum	17 (34.7%)	45 (33.3%)	χ^2^ = 0.03	0.863
Surgical approach			χ^2^ = 0.470	0.791
ACCF	9 (18.4%)	30 (22.2%)		
Laminoplasty	28 (57.1%)	70 (51.9%)		
Laminectomy	12 (24.5%)	35 (25.9%)		

The Chi-square test between minor trauma and post-operative outcomes showed that patients with a history of cervical minor trauma were more prone to poor prognosis (Table 2). In addition, patients with minor trauma had a lower pre-operative JOA score and a higher proportion of intramedullary signal changes (Table 3). The percentage of patients with cervical instability was also higher in patients with minor trauma, although there was no statistically significant difference.

**Table 3:** Comparison of characteristics of patients with and without minor trauma.

Characteristics	OPLL with minor trauma (n = 55)	OPLL without minor trauma (n = 129)	test value	P value
Instability of cervical spine	8 (14.5%)	8 (6.2%)	χ2 = 3.381	0.066
T1 hypointensity on MRI	34 (61.8%)	30 (23.3%)	χ2 = 25.278	**<0.001**
T2 hyperintentsity on MRI	51 (92.7%)	89 (69.0%)	χ2 = 11.939	**0.001**
Pre-JOA score	9.02±1.800	9.84±1.708	Z = -3.097	**0.002**
Post-JOA score	13.13±1.645	15.28±1.803	Z = -6.606	**<0.001**

The result of multivariate stepwise logistic regression showed that a longer duration of symptoms, T1 hypo-intensity intramedullary changes on MRI and a history of cervical minor trauma were significant risk factors of a poor neurological outcome in terms of JOA recovery ratio (Table 4).

**Table 4:** Stepwise logistic regression for lower recovery ratio of JOA score.

Measure	Odds Ratio	95% confidence intervals	P value
Duration of symptoms	1.023	1.012–1.035	**<0.001**
T1 hypointensity on MRI	4.544	1.995–10.352	**<0.001**
Minor trauma	2.573	1.123–5.897	**0.025**

Procedure-related complications included hematoma in 2 cases, cerebrospinal fluid (CSF) leakage in 6 cases, and C5 palsy in 13 cases. The neurological dysfunction in the two patients with hematoma was relieved after emergency operation. CSF leakage occurred after a dural tear during the operation due to tight adhesion to the dura or ossification of the dura. Most cases of CFS leakage were cured in a week after symptomatic treatment including drainage and local pressure dressing. C5 palsy developed in 8 hours postoperatively and was recovered in most cases in two months after conservative treatment.

## Discussion

Our study indicated that factors including age at operation, the duration of symptoms, diabetics, signal changes on MRI, transverse area of the spinal cord, cervical kyphosis, a history of cervical minor trauma and a lower preoperative JOA score were associated with a poor post-operative neurological outcome in term of JOA recovery ratio. The result of our multivariate stepwise logistic regression suggested that, among all these factors, a long duration of symptoms, T1 hypointensity on MRI and a history of cervical minor trauma were was significant predictive indicators of poor surgical outcome.

The duration of symptoms reflects the length of the course of myelopathy, and affects the severity and progression of the disease due to chronic compression by the ossified mass. The longer the spinal cord is compressed by the ossified posterior longitudinal ligament, the greater possibility of irreversible injury might exist. Studies on the outcome of cervical spondylotic myelopathy[15] [16] demonstrated that the duration of myelopathy was a significant factor related to the postoperative prognosis. A similar situation existed in patients with OPLL. Patients with unsatisfactory surgical outcomes for OPLL were observed to have a longer duration of symptoms in our study.

The association between the surgical outcomes of cervical compressive myelopathy and intramedullary signal intensity changes on MRI has long been a clinical concern[17]. Ramanauskas et al [18] divided myelomalacia into early, intermediate and late stage, saying that early/intermediate stage patients were characterized by spinal cord edema and cystic necrosis of the central gray matter, which were often represented by hyper-intensity changes on T2-weighted images, while late-stage patients were characterized by central cystic degeneration, syrinx formation and atrophy, which were often represented by hyper-intensity changes on T2-weighted sequences, and hypo-intensity changes on T1-weighted images. Ohshiro et al [19] concluded that the signal pattern of T1-isointensity/T2-hyperintensity changes indicated edema, gliosis, and a mild loss of nerve cells in the gray matter, and that the signal pattern of T1-hypointensity/T2-hyperintensity changes indicated myelomalacia, necrosis and spongiform change in the gray matter. In cases of CSM, patients with altered signal intensity on both T1WI and T2WI demonstrated a worse postoperative prognosis as compared with those only with hyperintensity on T2-weighted images[20–25]. A similar phenomenon was found to exist in cases of OPLL in this study. Our multiple regression analysis confirmed that hypointensity on T1WI was a significant risk factor of a poor outcome. But this does not mean that we can underestimate signal changes on T2WI. Both changes on T1WI and T2WI reflect pathological damages to the spinal cord, which may become irreversible with the progression of ossification and prolonged compression[26,27]. Thus, surgical intervention should be considered before the advent of signal intensity changes on MRI.

Minor Trauma caused by low-energy injuries including fall, whiplash injury, or strike with blunt objects would also result in poor prognosis. Although these minor traumas may not lead to bony fractures or dislocations as those high-energy traumas, they may still cause acute cord injuries[28–31]. A more common presence of spinal cord signal changes on MRI in patients with minor trauma revealed a worse pre-operative neurological status, which may be less sensitive to the surgical treatment. It was found in ours study that preoperative JOA scores in patients with minor traumas were relatively lower than those in the other patients, which is in consistence with the literature available. Although cervical instability was not significantly associated with the history of minor trauma, we cautiously suggest that sustained irritation caused by unstable discs and ossification should not be ignored. To prevent progressive deterioration of the neurological function, cervical decompressive surgery should be performed as soon as possible in patients with neurological deficits.

Although the duration of symptoms and the signal intensity are significant risk factors of poor prognosis in patients with OPLL, they are not the only factors associated with the surgical outcome.

Age may affect the recovery rate due to multiple factors. Age-related degeneration of motor neurons and myelinated fibers in the spinal cord may make elderly patients more vulnerable. In addition, general degeneration associated with the normal aging process and increased risk of underlying dieases also have negative influence on the recovery[7].

Severe diabetes will damage the peripheral nerves, and the central nervous system as well [32]. Diabetic patients are more likely to develop abnormal spinal cord changes including infarction, demyelination, atrophy and softening of the posterior column[33,34]. If the nervous system is directly damaged by diabetes, the outcome of decompression will not be satisfactory.

The severity of spinal cord compression is usually measured by the transverse area and compression ratio. Ohshio et al [19] suggested that morphologic changes of the spinal cord are sometimes associated with pathologic severity and may affect the postoperative functional improvement. Li et al [35] confirmed that decreased cross-sectional area of the spinal cord reflects compression-induced atrophy and severity of compression. In severe cases of OPLL, both the anteroposterior and transverse diameters of the spinal cord could be decreased due to massive compression and atrophy of the spinal cord. In such cases, the compression ratio (anteroposterior diameter / transverse diameter) may not be necessarily decreased. For this reason, the transverse area may be a more sensitive reference than the compression ratio[36].

Kyphotic patients often present with myelopathy because of increased stress on the ventral spinal cord, which adversely affects the spinal cord vasculature and is most likely to cause local ischemia[37,38]. Sun et al [27] confirmed that OPLL patients with kyphotic alignment were more likely to present intramedullary spinal cord changes on MRI and have a poor neurological outcome.

There exist controversies over the surgical strategies for OPLL[39,40]. Anterior resection of the ossified ligament is a radical surgical option for direct decompression[41]. Previous studies [42] have proved that anterior decompression can achieve satisfactory outcomes and therefore is considered as the primary option. Posterior decompression is an alternative option for indirect decompression by enlarging the spinal canal. Sun et al [43] suggested that the anterior approach could provide a more radical decompression in patients with severe OPLL by direct removal of the compressive mass, while in patient with mild OPLL, both the anterior approach and the posterior approach could get satisfactory surgical outcomes.

## Conclusion

The duration of symptoms and T1 hypo-intensity on MRI are highly predictive of the outcome of patients undergoing surgical treatment for OPLL. Other factors, including age at operation, a history of diabetes, the preoperative JOA score, the transverse area of the spinal cord and T2 hyper-intensity on MRI, are also closely correlated with the prognosis of OPLL. As persistent cord compression and the potential risk of disease progress may lead to a treatment failure, an understanding about the importance of predictive factors can help surgeons consider the indications of surgical treatment and evaluate the timing of surgery.

## Supporting Information

S1 File Patients' data. (XLSX)

S2 File Assignment description. (DOCX)
