# Supplementary material for: Clinical and Imaging Predictors of Surgical Outcome in Multilevel Cervical Ossification of Posterior Longitudinal Ligament: An Analysis of 184 Patients
Source: PLoS One. 2015 Sep 1;10(9):e0136042. doi: 10.1371/journal.pone.0136042 (PMC4556618; doi:10.1371/journal.pone.0136042)
Supplement: S2 File — (DOCX) [file pone.0136042.s002.docx]

| Factors | Assignment |
| --- | --- |
| Sex | Male=1;Female=0 |
| Age at operation | <45=1,45~=2,55~=3,65~=4 |
| Duration of symptom | <1=1,1~=2,3~=3,5~=4 |
| Diabetes | Yes=1，No=0 |
| Smoking | Yes=1，No=0 |
| Period of follow-up |  |
| Pre-op JOA score |  |
| Surgical approach | ACCF=1，Laminoplasty=2，Laminectomy=3 |
| Cervical alignment | Lordosis=1, Kyphosis=0 |
| K-line | K-line(+)=0,K-line(-) |
| Type of OPLL | Continuous type=1,Segmental type=2,  Mixed type=3,Circumscribed=4 |
| Shape of OPLL(Sagittal) | Hill=0,Plateau=0 |
| Shape of OPLL （transverse） | symmetrical=0，asymmetrical=1 |
| Occupying ratio | <40=1,40~=2,50~=3,60~=4,70~=5 |
| Transverse area of spinal cord | <30=1,30~=2,50~=3,70~=4 |
| Compression ratio of SC | <20=1,20~=2,30~=3 |
| Instability of cervical spine | Yes=1,No=0 |
| Compression levels | 连续变量 |
| Double-layer sign | Yes=1,No=0 |
| Simultaneous compression of hypertrophic ligamentum flavum | Yes=1,No=0 |
